# Human Blood Bacteriome: Eubiotic and Dysbiotic States in Health and Diseases

**DOI:** 10.3390/cells11132015

**Published:** 2022-06-23

**Authors:** Kanokphong Suparan, Sirawit Sriwichaiin, Nipon Chattipakorn, Siriporn C. Chattipakorn

**Affiliations:** 1 Immunology Unit, Department of Microbiology, Faculty of Medicine, Chiang Mai University, Chiang Mai 50200, Thailand; kanokphong.su@cmu.ac.th; 2 Neurophysiology Unit, Cardiac Electrophysiology Research and Training Center, Faculty of Medicine, Chiang Mai University, Chiang Mai 50200, Thailand; sirawit.sriwichaiin@cmu.ac.th (S.S.); nipon.chat@cmu.ac.th (N.C.); 3 Center of Excellence in Cardiac Electrophysiology Research, Chiang Mai University, Chiang Mai 50200, Thailand; 4 Cardiac Electrophysiology Unit, Department of Physiology, Faculty of Medicine, Chiang Mai University, Chiang Mai 50200, Thailand; 5 Department of Oral Biology and Diagnostic Sciences, Faculty of Dentistry, Chiang Mai University, Chiang Mai 50200, Thailand

**Keywords:** blood, microbiome, bacteriome, eubiotic, dysbiosis

## Abstract

The human gut microbiome is acknowledged as being associated with homeostasis and the pathogenesis of several diseases. Conventional culture techniques are limited in that they cannot culture the commensals; however, next-generation sequencing has facilitated the discovery of the diverse and delicate microbial relationship in body sites and blood. Increasing evidence regarding the blood microbiome has revolutionized the concept of sterility and germ theory in circulation. Among the types of microbial communities in the blood, bacteriomes associated with many health conditions have been thoroughly investigated. Blood bacterial profiles in healthy subjects are identified as the eubiotic blood bacteriome, whereas the dysbiotic blood bacteriome represents the change in bacterial characteristics in subjects with diseases showing deviations from the eubiotic profiles. The blood bacterial characteristics in each study are heterogeneous; thus, the association between eubiotic and dysbiotic blood bacteriomes and health and disease is still debatable. Thereby, this review aims to summarize and discuss the evidence concerning eubiotic and dysbiotic blood bacteriomes characterized by next-generation sequencing in human studies. Knowledge pertaining to the blood bacteriome will transform the concepts around health and disease in humans, facilitating clinical implementation in the near future.

## 1. Introduction

Nowadays, the terms “microbiome” and “microbiota” are used interchangeably; however, they are two different terms. The microbiota is an intricate ecosystem of microorganisms, whereas the microbiome is a collection of the genomes of the microorganisms in that community [1]. The eubiotic state or eubiosis is referred to microbiota that provide the host with health benefits. On the contrary, the dysbiotic state or dysbiosis is defined as changes in the proportion and/or taxa of microorganisms that deviate from a eubiotic profile. Additionally, the dysbiotic microbiota are not able to provide the host with the full range of beneficial functional properties [2]. The disturbance of this ecosystem could induce pathological consequences, especially persistent low-grade chronic inflammation, which may predispose long-term illness in humans [3,4].

Human blood, formerly recognized as a sterile component, was discovered to contain genetic materials of the blood microbiome, which are related to chronic inflammatory diseases [5]. The presence of live microbes in blood could potentially lead to sepsis. However, Potgietor et al. (2015) proposed the term atopobiosis to indicate the presence of microorganisms within the blood rather than in their normal habitat. This term indicates that the translocated microbes may exist latently within the circulation in a harmless state of dormancy and may be stimulated to initiate the immune response under suitable conditions [5]. Cell-wall deficient forms of bacteria and fungi recognized by the term L-form are good candidates for this phenomenon. When microbes are exposed to oxidative stress or cell-wall synthesis inhibitors, they are capable of shedding immunogenic components such as lipopolysaccharides (LPS) to become the L-form, which has less immunogenicity as a survival strategy of immunologic evasion [6,7,8]. The L-forms harvested from human blood are membrane-bound structures that range in size from 100 to 500 nm, similar to extracellular vesicles (EVs) [9]. Extensive exploration of blood microbiota by conventional culture methods was not effective due to the fastidiousness of each microbe [7,10]. Although methods of cell-wall induction were developed to stimulate L-form microbes, the methods involve delicate and time-consuming procedures. Currently, however, the availability of next-generation sequencing techniques for the detection and identification of blood microbiomes in humans gradually enabled the construction of the eubiotic and dysbiotic blood microbial profiles in both the healthy and diseased conditions. The 16s ribosomal RNA (rRNA) genes have been used to identify bacteria and archaea, and internal transcribed spacers have been used to identify fungi [11]. Nevertheless, it has not proved possible to distinguish whether those genetic materials belonged to viable or dead microorganisms or microbial cell-free nucleic acids [12]. Thereby, the blood microbiome may be an appropriate term at the present time instead of blood microbiota, which is still controversial. Most studies into the blood microbiome focused on the bacteriome, and the archeome, virome, and mycobiome were scantly explored [13,14,15].

Therefore, this review aims to summarize the eubiotic and dysbiotic profiles of the blood bacteriome, as characterized by next-generation sequencing. We also discussed the current understanding of those in human studies in the context of health and disease. The original articles were published in the PubMed database before November 2021, and the relevant articles were included in this review. The search terms “blood microbiome”, “blood microbiota”, and “circulating microbiome” were used to search the articles. A comprehensive summary of the human blood microbiome was reviewed elsewhere [16].

## 2. Profiles of the Eubiotic Blood Bacteriome

The eubiotic blood bacteriome in this review is defined by venous blood bacteriome profiles found in healthy adults, whose ages were described as being between 20 and 65 years in the selected articles. Table 1 summarizes the eubiotic profiles of blood bacteriome in healthy humans. The majority of the studies assessed the profiles by amplicon-based sequencing of 16s rRNA genes; a few studies used shotgun sequencing of bacterial DNA and RNA sequencing (RNA-seq). The eubiotic bacteriome at phyla level included Proteobacteria (9–99%) [17,18], Actinobacteria (0.01–76%) [19,20], and Firmicutes (1–64%) [21,22]. Those three phyla constitute over 70% of the eubiotic blood bacteriome, while Bacteroidetes, Fusobacterium, Cyanobacteria, Verrucomicrobia, and Acidobacteria are the minority group. Both the shotgun sequencing of bacterial DNA and the RNA-seq corroborated the parallel eubiotic blood profiles to the profiles evaluated by the amplicon-based sequencing [15,17,20,23].

The type of blood specimen may influence the differential abundance of the blood bacteriome between studies. Sex and geographic region might also be independent factors affecting the profiles of bacteriomes, as summarized in Table 1. The types of blood specimens in the studies of blood bacteriomes can be divided into three groups: (1) whole blood, (2) blood cell components, and (3) non-blood cell components. Specimens of the blood cell components in the studies included leukocytes and erythrocytes; leukocytes included peripheral blood leukocytes (buffy coat), peripheral blood mononuclear cells (PBMC), and neutrophils. Specimens of non-blood cell components are serum, plasma, and EVs. Whole blood may be the most representative specimen for the term blood bacteriome because it consists of all elements of the blood. The most abundant phylum of whole blood bacteriome in several studies was Proteobacteria, followed by Firmicutes and Actinobacteria [13,14,21,23,24,25,28]. Unexpectedly, Blood cell components may favor Proteobacteria as they occupied the highest proportion of bacteriome among types of the blood specimens [22,24,25,28,29,30,31,32]. However, the blood bacteriome profile from the specimens containing blood cells may be influenced by the bacterial cell-free DNA that is being removed by the innate immune response of leukocytes and circulating DNase; therefore, it may not reflect the natural ecology of the blood bacteria [48]. Thus, a number of studies preferred to use non-blood cell specimens to represent their blood bacteriome.

EVs are plasma-membrane-bound vesicles secreted by human cells and also bacteria into circulation. They could contain genetic materials that might convey crucial biological information [49]. The bacteriome of plasma or serum might be represented by EVs and bacterial cell-free circulating DNA, which might not be eliminated by the circulating DNase. In order to eradicate the effect of cell-free circulating DNA, the samples could be treated with additional DNase as a first step before they underwent extraction. Emery et al. (2020) pointed out that there was an alteration among the most abundant phyla in which Firmicutes could overcome Proteobacteria and Actinobacteria on the use of DNase [21]. Lee et al. (2020) and Chang et al. (2021) reported that Firmicutes were the most abundant phylum in the circulating bacterial EVs [44,45]. On the contrary, other studies found that the majority of the EVs comprised Proteobacteria instead of Firmicutes [42,43,46]. The total numbers of healthy subjects in the latter studies were far fewer than those in those two former studies. In summary, all of these findings suggested that Firmicutes were the most abundant phyla among the EV bacteriome [44,45]. Thus, Firmicutes might be representative of potential cell-wall deficient bacteria outside leukocytes. Presumably, most bacterial cell-free circulating DNA might belong to Proteobacteria and Actinobacteria. However, the observation of the selectivity of microorganisms in each type of blood specimen is inexplicable and needs further validation by experimental studies to give insight into the mechanism. Interestingly, sample preparation processes other than the DNase pretreatment could also influence the composition of bacteriome profiles [13,14,21]. Panaiotov et al. (2018, 2021) showed that whole blood pretreated by the stimulated process prior to DNA extraction yielded similar bacterial compositions at the phyla level compared with ordinary samples [13,14]. Nonetheless, several genera had an increase in the number of taxonomic reads after the resuscitation process [13,14]. This evidence leads to the possibility that some circulating bacteria might have the ability to persist and proliferate within the blood samples according to the resuscitation process.

## 3. Profiles of Dysbiotic Blood Bacteriome

Knowledge regarding germ theory has increased vastly over many years. Since the discovery of next-generation sequencing, it has become apparent that the pathogenesis of infectious diseases might not account for only one kind of microbes but that various microorganisms living together as microbiota could influence the infection [50]. Beyond that, dysbiotic microbiota could also potentiate non-communicable diseases [51,52]. In this review, the dysbiotic venous blood bacteriome is described as the profile of altering bacterial composition in a condition group that deviated from an assigned control group in the same population. Many related conditions that exhibit blood dysbiosis are categorized into five clusters, including infection-related diseases (Table 2), age-related metabolic diseases (Table 3), oral/gastrointestinal/hepatobiliary diseases (Table 4), neurological disorders (Table 5), and immune-mediated diseases (Table 6).

### 3.1. Infection-Related Diseases and Profiles of Blood Dysbiosis

Several studies reported that the blood bacteriome was altered in cases of human immunodeficiency virus (HIV) infection (Table 2) [49,51,53]. HIV infection was shown to develop blood dysbiosis as indicated by an increase in Proteobacteria and a decrease in Actinobacteria and Firmicutes [40]. Gut bacterial translocation as a result of gut epithelial disruption from mucosal immune defects might be responsible for blood dysbiosis [43]. Although HIV patients treated well with combined antiviral therapy (cART) could show improved immune status and diminished viral load, a non-nucleotide-reverse-transcriptase-inhibitor-based regimen, as well as a protease-inhibitor-based regimen, might not ameliorate the disruption of the gut epithelial barriers [38]. In addition, those treatments might further damage the gut barrier [69] and lead to persistent gut bacterial translocation [38]. Another study found that the presence of *Massilia* and *Haemophilus* in the blood of well-treated HIV patients with cART may induce the production of proinflammatory cytokines from PBMC, leading to potentiation of chronic systemic inflammation in the long term [53]. Additionally, cART might modify the blood bacteriome by an upsurge in *Staphylococcaceae*. *Staphylococcus* could be implicated in autoimmune diseases in well-treated HIV patients as a consequence of the development of autoreactive B cells and auto-antibody production [40]. The findings from these studies suggested that HIV infection could affect the blood bacteriome, and antiretroviral therapy might be associated with gut barrier disruption. These could result in chronic inflammation and altered autoimmune status.

Sepsis is an infection accompanied by systemic immune dysregulation, and sepsis with the presence of multiorgan dysfunction indicates septic shock. Various septic models depicted different aspects of blood bacterial profiles (Table 2) [19,28,54,70,71]. Some studies showed that blood dysbiosis of septic patients might have a higher proportion of Proteobacteria or Bacteroidetes [19,28]. Two previous studies showed a profile of septic patients might show a reduction in Actinobacteria [19,28]. Nevertheless, a rise in *Agrococcus* in Actinobacteria may be involved in the progression of sepsis [28]. An infectious site could cause changes in blood dysbiosis. For instance, Bacteroidetes, the most abundant phylum of the lung microbiome, was increased in the blood of animal models with lung injuries [70,71]. In some pre-term newborns who had a peripherally central catheter inserted, a central line bloodstream infection developed [54]. Even though the blood profiles of septic adults may not apparently specify a single causative pathogen of sepsis, the entire blood bacteriome of the septic pre-term newborns shifted to bacteria of catheter’s biofilm as a suspected source of sepsis [54]. The bacteria recognized as pathogens included *Proteus* and *Staphylococcus* [54]. The dissimilarity of the septic blood bacteriome between two age groups may imply that the blood bacteriome of the pre-term newborns may be vulnerable to invasion by pathogens. Moreover, the complexity of the blood bacteriome in the adults may involve distinct pathogenesis of sepsis when compared with the pre-term newborns.

There are two studies that mentioned that the blood bacteriome in pregnant women with pre-term delivery differed from those who had term delivery at two time points, gestational age 15–16 weeks (mid-trimester) and labor stage (9 months) (Table 2) [55,56]. In the first study, the pregnant women who had an increase in Proteobacteria and Actinobacteria together with a reduction in Firmicutes and Bacteroidetes in the mid-trimester of pregnancy tended to have pre-term delivery [56]. The second study indicated that, at the labor stage, the blood bacteriome of pregnant women with pre-term delivery might have an upsurge in Firmicutes and Bacteroidetes along with a decrease in Proteobacteria [55]. The contrasting profiles could be accounted for by the different time points of blood collection in both studies. The current knowledge is limited by a lack of parallel comparison of blood bacteriome profiles in different timepoints of pregnancy between those with term and pre-term delivery. In addition, there are several well-known factors that could affect the gut microbiota during pregnancy. Those factors in mothers include immunologic changes, underlying diseases, genetics, and diets [72]. They not only impact the composition of blood bacteriome in mothers but also affected on gut microbiota and blood microbiome of the newborn.

### 3.2. Age-Related Metabolic Diseases and Profiles of Blood Dysbiosis

The incidence of metabolic disease has been increasing in late adults globally due, in many cases, to a sedentary lifestyle [73]. Gut dysbiosis, together with the translocation of gut microbial constituents into the blood, could cause metabolic diseases as a result of persistent chronic low-grade systemic inflammation and insulin resistance [74]. Interestingly, bacterial 16s rRNA gene concentration in the blood of healthy subjects tended to increase with age [75]. Moreover, the high levels of blood bacterial 16s rRNA gene concentration may be associated with a slight disturbance in clinical blood parameters, including higher glucose levels, insulin levels, and free fatty acids levels accompanied by higher leukocyte counts, including both neutrophils and lymphocytes, in healthy late-adults when compared with healthy young adults [75].

Many studies focused on the various aspects of relationships between blood dysbiosis and type 2 diabetes mellitus (T2DM), especially as regards the profiles and mechanistic insights (Table 3) [18,22,30,57,58,76]. Amar et al. (2011) reported that healthy subjects whose blood contained a lower amount of Proteobacteria, as well as a higher amount of Actinobacteria, may have a reduced chance of the development of T2DM in the future [22]; however, higher blood 16s rRNA concentration in a healthy person might lead to T2DM [22]. Blood dysbiosis in patients with T2DM was characterized by a reduction in Rhodospirillales together with Myxococcales [18]. In addition, *Bacteroides* might be a protective factor for T2DM, while *Sediminibacterium* might be a risk factor for T2DM [18]. Among patients with morbid obesity, Anhe et al. (2020) found that *Enterobacteriaceae*, specifically *Escherichia-Shigella*, and *Neisseriaceae* were increased in the patients who had T2DM [57]. However, Massier et al. (2020) observed the differences at only the genus level; patients with morbid obesity and T2DM tended to have a higher level of *Tahibacter* with lower levels of *Delftia*, *Lactobacillus*, and *Lactococcus* when compared to subjects with morbid obesity without T2DM [58]. In patients with obesity, blood dysbiosis was likely to show an increase in Propionibactereles, Sphingomonadales, and Norcardioides [30]. In addition, there was a higher bacterial diversity as well as a higher proportion of Proteobacteria in liver bacteriome [30]. It is possible that an increase in the diversity of liver bacteriomes might be a consequence of the rise in circulating Proteobacteria from gut dysbiosis in obesity [76]. Adipocytes inoculated with bacteria DNA could produce inflammatory cytokines and other soluble anti-bacterial molecules, such as tumor-necrosis-factor-α, interleukin-6, C-reactive protein, and LPS-binding proteins [58]. Consequently, chronic systemic inflammation as pathogenesis of obesity and T2DM may be associated with the presence of bacterial DNA in blood and adipocytes. Intriguingly, mesenteric adipose tissues showed a high concentration of *Bacteroides*, most of which were gut commensals, and had a higher diversity of bacteriomes than blood, liver tissue, and other adipose tissues at different sites [57]. These findings suggested that mesenteric adipose tissue could harbor gut-translocating microbes, and the bacteriome of this tissue could potentiate persistent chronic systemic inflammation, finally resulting in metabolic aberrance. Furthermore, the liver contained a higher concentration of 16s rRNA bacterial genes than blood, indicating the crucial role of the liver as a filter of the blood bacteriome drained from both hepatic arteries and portal veins [57]. Moreover, an increase in 16s rRNA bacterial genes in the liver could give rise to fatty liver and chronic steatohepatitis [30]. The enigmatic interactions among blood, liver, and adipose bacteriomes should be investigated further to elucidate the pathophysiology behind the metabolic disease.

Hypertension is one of the most common age-related metabolic diseases affecting older adults worldwide. Blood dysbiosis in patients with hypertension compared with healthy controls might be characterized by an upsurge in Proteobacteria but a lower abundance of Firmicutes and Bacteroidetes [41]. Additionally, *Staphylococcus* might be a protective factor for hypertension, while either *Acinetobacter* or *Sphingomonas* might be a risk factor for hypertension [41]. Another study only detected the differences at only genus levels, specifically that a rise in *Streptococcus*, *Lactobacillus*, *Parabacteroides*, and *Helicobacter* and a decrease in *Stenotrophomonas* and *Turicibacter* might represent the dysbiotic blood profiles of hypertension [27]. The FAPROTAX database indicated that the blood bacteriome of both healthy subjects and hypertensive patients had similar patterns to bacterial patterns found in gastroenteritis, diarrhea, and pneumonia [27]. These findings may lead to the hypothesis that the blood bacteriome could increase in diversity during an inflammatory state of either gut or lung in which epithelial barriers might be disrupted, resulting in the translocation of residential microbes into the circulation.

Not only subjects with coronary heart diseases but also those with congenital heart and valvular heart diseases had changes in the blood bacteriome (Table 3). Patients with cardiac diseases had an increase in concentrations of 16s rRNA bacterial genes [15,23]. Amplicon-based sequencing of the blood bacteriome of patients with cardiac diseases demonstrated an increase in Proteobacteria with a reduction in Firmicutes [23]. The same blood samples of three patients with each type of cardiac disease were reinvestigated by shotgun sequencing. On the contrary, those results showed that Proteobacteria were decreased, but Actinobacteria were increased [15]. These results may point out that the platform of next-generation sequencing techniques might impact the taxonomic assignment of the blood bacteriome. In addition to coronary heart disease, blood dysbiosis in patients with myocardial infarction, when compared with controls with high cardiovascular risk, showed a lower proportion of *Norcardiaceae* and *Aerococcaceae* [59]. Both families of bacteria are cholesterol-degrading microbes, which could potentially prevent cardiovascular diseases such as atherosclerosis and ischemic heart diseases [59].

Other diseases, including chronic kidney disease and cerebrovascular accidents, also showed blood dysbiosis (Table 3). Dysbiotic blood of chronic kidney disease, but not end-stage, was characterized by an increase in Proteobacteria, especially *Enterobacteriaceae* and *Pseudomonadaceae*. Moreover, a higher proportion of Proteobacteria in blood was shown to potentially deteriorate kidney function as observed in a reduction in glomerular filtration rate [31]. Cerebrovascular accident, particularly ischemic stroke, is potential morbidity for patients with poorly controlled metabolic syndrome. The diversity of the blood bacteriome between patients with ischemic stroke and healthy controls was definitely distinct specifically due to an increase in Proteobacteria and a decrease in Firmicutes [45]. The patients who had a higher abundance of *Aerococcaceae* together with *Microbacteriaceae* but a decline in *Ruminococcaceae* tended to have good clinical outcomes [45].

In summary, an upsurge in Proteobacteria in the blood of various diseases in older adults indicates a general concept of blood dysbiosis in age-related metabolic diseases. It would also be interesting to investigate further how the cholesterol-degrading *Aerococcaceae* could benefit patients with vascular diseases such as ischemic heart disease and ischemic stroke.

### 3.3. Oral, Gastrointestinal, and Hepatobiliary Diseases and Profile of Blood Dysbiosis

The disturbances of the oral bacteriome due to various etiologies could affect the blood bacteriome (Table 4). For instance, chronic smoking, which causes a disturbance in the oral bacteriome, showed a correlation with an increase in *Streptococcus* in the blood [53]. Most of the species identified were oral commensals, including *Streptococcus parasanguinis*, *Streptococcus australis*, and *Streptococcus oligofermentans* [53]. Furthermore, dysbiotic blood of patients with periodontitis might be characterized by a decline in Candidatus Saccharibacteria [21]. Emery et al. (2020) showed that 70% of the blood bacteriome in both healthy subjects and patients with periodontitis were similar to bacteria that belonged to the Human Oral Microbiome database [21]. This information suggested that the oral bacteriome might be the source of the blood bacteriome.

Dysbiotic human blood bacteriome associated with stomach cancer might be characterized by a rise of *Haemophilus*, *Acinetobacter*, and *Bacteroides,* but a reduction in *Comamonas*, *Sphingomonas*, and *Pseudomonas*. The presence of *Enterococcus* in the blood of patients with stomach cancer might be associated with the progression of cancer as indicated by higher staging, deep invasion, and lymphatic metastasis. However, the increased abundance of *Haemophilus* in the blood of some patients might be a compensatory effect that aims to prevent lymphatic metastasis [35].

The ecosystem of the colon harbors the majority of gut microbiota. Colon resection for the treatment of colon cancer could downsize this community and induce dysbiosis of the remaining gut, as indicated by an increase in Proteobacteria and a decrease in Actinobacteria [77]. A decline in the concentration of the16s rRNA bacterial gene in the blood of post-colon-resection patients and a change in the blood bacteriome towards Proteobacteria, together with a reduction in Actinobacteria, could substantiate the concept that the gut may be the major source of blood bacteriome [60]. In another study, patients with colon cancer, most of whom were post-operative colon resection, were randomly treated with either chemotherapy alone (CT) or CT with adjunctive immunotherapy (dendritic cell/cytokine-induced killer cell, DC-CIK) [61]. After the second cycle of the drugs, both CT and CT with DC-CIK patients showed a decrease in the concentration of 16s rRNA bacterial gene in blood [61]. That evidence suggested that pathologic lesions of the colon might behave as a bypass that facilitates the translocation of the gut bacteriome into the blood. In addition to colon cancer, inflammatory bowel disease, Crohn’s disease, and ulcerative colitis have prominent gut dysbiosis and chronic gut inflammation [78]. These findings indicate that inflammatory bowel diseases might result in changes in the blood bacteriome. However, a characterization of the blood bacteriome in patients with treated inflammatory bowel diseases was similar to the blood bacteriome of healthy controls [46]. Blood dysbiosis in active inflammatory bowel disease requires further investigation to explore the hypothesis.

The pancreatobiliary system includes the pancreas, gall bladder, and a delicate structure of ducts that drain multiple enzymes from the pancreas, and bile acids from the gall bladder and liver, into the small intestine for food digestion. Structural abnormality, duct obstruction, and existing inflammation within the system could make it susceptible to an infection from the gut bacteriome [79,80,81]. In acute pancreatitis, Bacteroidetes were increased in both the whole blood and neutrophils of the patients when compared with healthy controls, while Actinobacteria were reduced [24]. This may be a consequence of nearby gut inflammation along with gut barrier disruption. An expansion or left shift of neutrophils in association with acute inflammation makes neutrophils a majority among the other types of white blood cells. The similarity of bacterial profiles between whole blood and neutrophils in cases of acute pancreatitis might be an example of blood bacteriome profiles in the systemic inflammatory response, in which neutrophil bacteriome could reflect blood bacteriome. In addition, the composition of the blood bacteriome in both patients with acute pancreatitis and healthy controls were similar to the gut microbiome, according to Human Microbiome Project [24]. These findings suggested that the blood bacteriome might primarily originate from the gut bacteriome in both the healthy condition and also in acute pancreatitis. Another study found that blood dysbiosis in biliary diseases, including biliary tract cancer, cholecystitis, and cholangitis, might be characterized by an upsurge in Clostridia but a decline in Gammaproteobacteria.

The liver receives its blood supply from both hepatic arteries, providing oxygen, and portal veins, providing the nutrients from the gut. Chronic hepatitis from any pathogenesis can turn into hepatic cirrhosis, in which the function of the liver ranges from compensatory status to decompensatory failure. Finally, cirrhosis can develop into liver cancer, particularly hepatocellular carcinoma. The physiologic role of the liver in the case of the blood bacteriome is questionable as to whether the liver could filter the blood bacteriome, and liver diseases might lead to changes in the composition of the blood bacteriome (Table 4). Alcoholic hepatitis, acute injury according to massive alcohol consumption, led to an increased concentration of the 16s rRNA bacterial gene in the blood, whereas Bacteroidetes were decreased [26]. The non-alcoholic fatty liver might be associated with a shifting of the blood bacteriome towards an upsurge in *Succinivibrionaceae* and a reduction in *Leukonostocaceae* [62]. Cirrhosis with compensated liver function might result in changes in the blood bacteriome towards an increase in Proteobacteria and a decrease in Firmicutes [34]. Decompensated liver function compared with normal liver function might lead to blood dysbiosis, as shown by a decline in Actinobacteria and Deinococcus–Thermus [63]. Additionally, a stepwise increase was shown in the concentration of the 16s rRNA bacterial gene from normal liver function to hepatitis B virus infection (HBV) with compensated liver function to HBV with decompensated liver function [63]. Proteobacteria, *Enterobacteriaceae* in particular, were raised in cirrhosis with compensated liver function; nonetheless, a lower proportion of *Enterobacteriaceae* was observed in HBV patients with acute decompensated liver function [17,63]. Interestingly, an increase in *Enterobacteriaceae* in a subgroup of patients with HBV and acute decompensated liver function resulted in a higher mortality rate than in the other groups [63].

Conclusively, the majority of blood bacteriomes may originate from the gut, particularly in the case of the colon, oral, and lung bacteriomes in which disruption of the epithelial barrier might instigate the translocation of microorganisms. Intriguingly, the liver may be responsible for blood bacterial filtration, Proteobacteria being the primary target for filtration by the liver. Therefore, the decline in liver function may result in blood dysbiosis in which the proportion of Proteobacteria in the blood might upsurge.

### 3.4. Neurological Disorders and Profiles of Blood Dysbiosis

The gut–brain axis depicts the impact of the gut microbiome on brain pathology. Blood is responsible for the transport of microbial constituents, particularly LPS and bacterial amyloid curli, from the gut to the brain. Those could disrupt the blood–brain barrier and initiate aberrant protein aggregation within the brain parenchyma, leading to neuroinflammation [82]. Blood dysbiosis of patients with untreated major depressive episodes might be characterized by a decline in Fusobacteria and Candidatus Saccharibacteria at the phyla level as well as an increase in *Janthinobacterium* and a reduction in *Neisseria* at the genus level (Table 5) [64]. Remarkably, this profile could revert following therapy with anti-depressive drugs, which increased the abundance of *Neisseria* and decreased *Janthinobacterium* [64]. In addition, the baseline profiles of the blood bacteriome in the untreated patients might predict drug responsiveness [64]. Patients with a higher proportion of Firmicutes, but a lower proportion of Proteobacteria and Actinobacteria, in the blood microbiome responded well to the drugs [64]. Patients with Schizophrenia had an upsurge in Planctomycetes and Thermotogae in the blood when compared with healthy controls and patients with bipolar disorder and amyotrophic lateral sclerosis [47]. Surprisingly, the blood bacteriome of patients with bipolar disorder and amyotrophic lateral sclerosis was similar to the blood bacteriome of healthy controls [47]. Therefore, the bacteriome may not be related to neurological impairment in some neurological disorders.

The blood bacteriome profiles of healthy subjects and the other neurological disorders were also similar to the gut and oral microbiome, according to Human Microbiome Project [47]. These findings suggested that the origin of the changes in the blood bacteriome in the case of neurological disorders may be from the gastrointestinal tract as in other groups of diseases. Blood dysbiosis in patients with Parkinson’s disease might be described by an increase in *Myroides*, *Isoptericola*, *Microbacterium*, *Cloacibacterium*, and *Enhydrobacter* and a decrease in *Limnobacter* [65]. In the blood of patients with multiple system atrophy (MSA), *Bacteroides were* increased, but *Leucobacter* was decreased. Additionally, the subtypes of MSA had different profiles in each subtype [66]. Cerebellar MSA, compared with parkinsonian, tended to have a higher abundance of *Acinetobacter*, while *Blastococcus* and *Bacillus* were decreased [66].

There is cautious evidence to show that food consumption and type of diet may be major confounders in blood bacteriome studies in neurological disorders, which therefore might not be directly caused by blood dysbiosis. On the contrary, blood dysbiosis might reflect the nutritional status and gut dysbiosis of the patients with mental disorders, which may cause a loss of appetite or a lack of ability to eat by themselves. Accordingly, the changes in gut microbial composition might shift the profiles of blood bacteriomes.

### 3.5. Immune-Mediated Diseases and Profiles of Blood Dysbiosis

Autoimmune diseases are chronic inflammatory diseases mediated by autoreactive B cells and autoreactive T cells against self-antigens, resulting in self-damage, which ranges from a specific organ to multiorgan systems. Numerous known or suspected autoimmune diseases have been shown to exhibit various changes in the blood bacteriome deviating from patterns found in healthy controls (Table 6). Systemic lupus erythematosus (SLE) is mediated by anti-nuclear factors and anti-double-stranded DNA antibodies. Surprisingly, a reduction in *Paenibacillus* in blood was concurrently observed in both SLE patients and their first-degree relatives when compared with healthy subjects [39]. Thereby, genetic factors may be related to this correlation. Another study showed that *Planococcus* was increased in SLE patients. Moreover, PBMC inoculated with *Planococcus* could produce significant levels of inflammatory cytokines, which might cause chronic inflammation in SLE [53]. A higher abundance of Cytophagia in the blood of patients with large-vessel vasculitis, for example, giant cell arteritis and Takayasu’s arteritis, could reflect the dysbiotic profiles when compared with healthy subjects [37]. The presence of *Staphylococcus* in blood might also play a role in the deterioration of Takayasu’s arteritis [37]. Blood dysbiosis in rheumatoid arthritis might be characterized by an upsurge in *Lachnospiraceae*, *Halomonas*, and *Shewanella*, while *Corynebacterium 1* and *Streptococcus* decreased. Anti-rheumatic drugs might cause a reversion of blood dysbiosis in rheumatoid arthritis by causing an increase in *Corynebacterium 1* and *Streptococcus* but a decrease in *Shewanella* [36]. However, the persistent rising of *Lachnospiraceae* after the treatment might indicate that those bacteria may be a compensatory effect of blood dysbiosis, and this increase might ameliorate the disease. In addition, an increase in *Pelagibacterium* together with *PARP9* mRNA levels might be a part of the pathogenesis of rheumatoid arthritis [32]. In cases of psoriasis, blood dysbiosis might be characterized by a decrease in Firmicutes and Fusobacteria [42]. Although some bacteria could induce autoreactivity, the profiles among those autoimmune diseases were diverse even within the same conditions. The role of the blood bacteriome in each disease requires further research.

In addition to the autoimmune diseases already discussed, asthma sufferers, patients with immune-mediated reversible obstructive airway disease, showed an increase in Bacteroidetes in the blood dysbiotic profiles [44,68]. The possible explanation may be that the lung bacteriome predominated by Bacteroidetes might translocate into the circulation during airway inflammation [70,71]. Long-term use of steroids used for the reversion of airway obstruction might also affect the blood bacteriome of patients with asthma, as indicated by decreases in *Staphylococcus* and *Rothia.* Furthermore, systemic steroids might cause an increase in *Prevotella 9*, *Intestinibacter*, *Lactobacillus*, and *Blautia* in the blood [44]. Blood dysbiosis in rosacea, chronic vascular and inflammatory skin disease, might be characterized by an upsurge in *Chromaticeae*, *Rheinheimera* in particular, and *Fusobacteriaceae* [67].

## 4. Limitations

This review comprehensively summarized the evidence of blood bacteriome in various conditions. However, there were still some limitations. The information included in this review was derived from the original articles that are restricted to the English language and are in the PubMed database. Some articles related to this topic published in other medical databases and other languages might be missed. In addition, analogous keywords used for article selection apart from the terms “blood microbiome”, “blood microbiota”, and “circulating microbiome”, might also influence the contents of this review. Importantly, the dysbiosis and eubiosis blood bacteriome profiles were formulated on results from next-generation sequencing. The blood bacteriome characterized by other techniques was not included.

## 5. Conclusions

**Figure 1:**
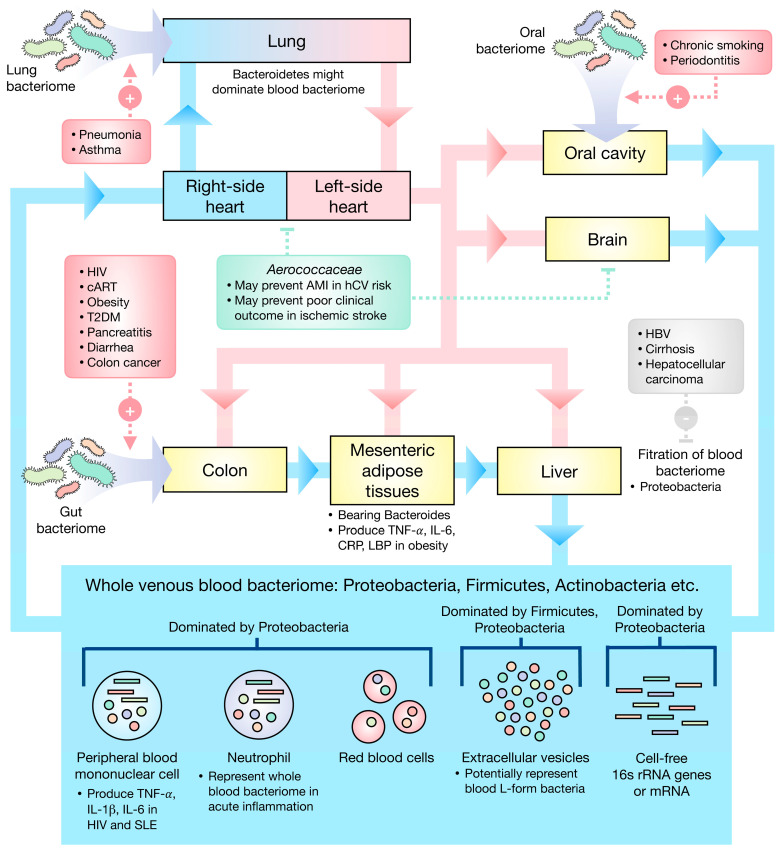
Schematic illustration of the current understanding of the blood bacteriome. (**Red box**) Gut, the colon in particular, oral, and lung bacteriomes may be the primary sources of the blood bacteriome, several factors potentially influencing the translocation of the bacteria. (**Gray box**) The liver could filter the blood bacteriome, especially Proteobacteria, and liver diseases may cause a deterioration of this function. (**Green box**) The Cholesterol-degrading *Aerococcaceae* may play a role in the amelioration of ischemic stroke and prevention of AMI. AMI—acute myocardial infarction, hCV—high cardiovascular risk, HIV—human immunodeficiency virus, cART—combined anti-retroviral therapy, T2DM—type 2 diabetes mellitus, HBV—hepatitis B virus infection, SLE—systemic lupus erythematosus, TNF—tumor necrosis factor, IL—interleukin, CRP—C-reactive protein, LBP—LPS-binding protein.

The eubiotic blood bacteriomes were found to be dominated by Proteobacteria, Firmicutes, and Actinobacteria. Gut, oral, and lung bacteriomes may be the primary sources of the blood bacteriome. Figure 1 presents the current understanding as regards human blood bacteriome.

Dysbiotic human blood bacteriome could be a consequence of cutaneous or mucosal barrier disruption as well as the inability of the liver to filter the blood bacteriome. Intriguingly, some diseases well-treated with appropriate medication showed a reversion of dysbiotic profiles to eubiotic profiles suggesting that blood dysbiosis may be a consequence of poorly controlled disease. Though the assumption of dysbiotic human blood bacteriome as a cause of various diseases seems to be reasonable, the cause–effect relationship between blood bacteriome and each disease has to be investigated to confirm the actual relations.

Taken together, the groundwork already completed regarding the characteristics of the blood bacteriome transformed the perceptions around germ theory and improved the comprehension of the blood bacteriome, further changing the concepts of health and disease in humans, resulting in clinical implementations in the near future.

## Figures and Tables

**Table 1:** Eubiotic Characteristics of the Blood Bacteriome in Healthy Humans.

Blood Specimen	Subjects n (M/F)	Age ^#^	Country	Hypervariable Region (V)	Taxonomic Database	Order of Relative Abundance at Phylum Level				Ref.
						First	Second	Third	Other	
DNA										
Whole blood	10 (9/1)	29.2 ± 11.26	India	V3	Greengenes	Proteobacteria	Firmicutes	Actinobacteria	NA	[23]
	12 (10/2)	29.2 ± 3.8	China	V3	RDP	Proteobacteria	Actinobacteria	Firmicutes	Bacteroidetes	[24]
	3 (2/1)	38.33 ± 20.98	UK	V3–V4	SILVA	Proteobacteria	Actinobacteria	Firmicutes	Bacteroidetes, Fusobacteria	[21]
	60 (18/42)	39.8 ± 9.5	Italian	V3–V4	NCBI	Proteobacteria	Actinobacteria	Firmicutes	Bacteroidetes	[25]
	19 (4/15)	39.89 ± 13.69	UK	V3–V4	SILVA	Firmicutes	Proteobacteria	Actinobacteria	Bacteroidetes, Fusobacteria	[21] ^†^
	20 (5/15)	41.9 ± 10.7	USA	V3–V4	Greengenes	Firmicutes	Proteobacteria	Actinobacteria	Bacteroidetes	[26]
	28 (14/14)	45 ± 12	Bulgaria	V3–V4	Greengenes	Proteobacteria	Firmicutes	Actinobacteria	Planctomycetes	[14] ^†^
	28 (14/14)	45 ± 12	Bulgaria	V3–V4	Greengenes	Proteobacteria	Firmicutes	Actinobacteria	Bacteroidetes, Cyanobacteria	[14] ^†,‡^
	23 (10/13)	59	Poland	V3–V4	RDP, Greengenes	Actinobacteria	Proteobacteria	Firmicutes	Bacteroidetes, Cyanobacteria	[19]
	28 (NA)	NA	China	V3, V4, V3–V4, V4–V5	Greengenes	Firmicutes	Bacteroidetes	Proteobacteria	Actinobacteria, Cyanobacteria	[27]
	5 (NA)	NA	China	V3	RDP	Proteobacteria	Actinobacteria	Firmicutes	Bacteroidetes	[28]
	28 (NA)	NA	Bulgaria	V3–V4	SILVA	Proteobacteria	Firmicutes	Actinobacteria	Planctomycetes, Armatimonadetes	[13] ^†^
	28 (NA)	NA	Bulgaria	V3–V4	SILVA	Proteobacteria	Firmicutes	Actinobacteria	Bacteroidetes, Fusobacteria	[13] ^†,‡^
Buffy coat	30 (9/21)	21 (18–53)	France	V3–V4	NCBI	Proteobacteria	Actinobacteria	Firmicutes	Bacteroidetes	[29]
	15 (15/0)	40 (25–68)	Denmark	V3–V4	NCBI, SILVA	Proteobacteria	Actinobacteria	Firmicutes	Acidobacteria, Bacteroidetes	[30]
	20 (7/13)	44 (39–53)	USA	V3–V4	SILVA	Proteobacteria	Bacteroidetes	Actinobacteria	Firmicutes	[31]
	26 (5/21)	46.2 ± 8.9	Spain	V3–V4	NCBI	Proteobacteria	Actinobacteria	Firmicutes	Bacteroidetes	[25]
	28 (NA)	47 ± 10	France	V1–V2	SILVA	Proteobacteria	Bacteroidetes	Actinobacteria	Firmicutes, Acidobacteria	[22]
Neutrophil	12 (10/2)	29.2 ± 3.8	China	V3	RDP	Proteobacteria	Actinobacteria	Firmicutes	Bacteroidetes	[24]
	5 (NA)	NA	China	V3	RDP	Proteobacteria	Actinobacteria	Firmicutes	Bacteroidetes	[28]
PBMC	14 (0/15)	50.48 ± 14.05	China	V3–V4	SILVA	Proteobacteria	Actinobacteria	Bacteroidetes	Deinococcus–Thermus, Firmicutes	[32]
Red blood cell	30 (9/21)	21 (18–53)	France	V3–V4	NCBI	Proteobacteria	Actinobacteria	Firmicutes	Bacteroidetes, Fusobacteria	[29]
Serum	24 (10/14)	27.8 ± 4.0	USA	V4	RDP	Firmicutes	Bacteroidetes	Proteobacteria	Fusobacteria, Actinobacteria	[33]
	201 (119/82)	57.6 ± 10.4	Korea	V3–V4	Greengenes	Firmicutes	Proteobacteria	Actinobacteria	Bacteroidetes, Verrucomicrobia	[34]
	24 (10/14)	63.9 ± 3.2	USA	V4	RDP	Firmicutes	Bacteroidetes	Proteobacteria	Actinobacteria, Fusobacteria	[33]
	13 (NA)	NA	China	V1–V2	RDP, Greengenes	Proteobacteria	Actinobacteria	Firmicutes	Deinococcus–Thermus, Bacteroidetes	[35]
	4 (NA)	NA	UK	V4	SILVA	Proteobacteria	Firmicutes	Bacteroidetes	Actinobacteria, Fusobacteria	[36]
	15 (NA)	NA	France	V3–V4	Greengenes	Proteobacteria	Bacteroidetes	Actinobacteria	Firmicutes, Gemmatimonadetes	[37]
Plasma	30 (9/21)	21 (18–53)	France	V3–V4	NCBI	Proteobacteria	Actinobacteria	Firmicutes	Bacteroidetes	[29]
	3 (2/1)	27 ± 3.46	India	Shotgun	MG-RAST/ SEED	Proteobacteria	Actinobacteria	Firmicutes	NA	[15]
	15 (15/0)	29 (24–33)	Italy	V3–V4	NCBI, SILVA	Proteobacteria	Actinobacteria	Firmicutes	Bacteroidetes	[38]
	19 (0/19)	34.2 ± 9.4	USA	V4	Greengenes	Proteobacteria	Fusobacteria	Actinobacteria	Firmicutes, Bacteroidetes	[39]
	16 (5/11)	38 (33–55)	USA	V4	NCBI, RDP	Proteobacteria	Firmicutes	Actinobacteria	Bacteroidetes	[40]
	18 (3/15)	38.6 ± 12.4	USA	V4	Greengenes	Proteobacteria	Actinobacteria	Firmicutes	Bacteroidetes, Cyanobacteria	[39]
	5 (0/5)	39.4 ± 10.3	UK	V4	SILVA	Proteobacteria	Actinobacteria	Firmicutes	Bacteroidetes	[20]
	150 (66/84)	48.13 ± 6.22	China	V6–V7	NA	Proteobacteria	Firmicutes	Actinobacteria	Bacteroidetes	[41]
	100 (64/36)	51.98 ± 8.05	China	V5–V6	NA	Proteobacteria	Bacteroidetes	Firmicutes	Actinobacteria	[18]
EVs	8 (5/3)	49.63 ± 15.16	Taiwan	V1–V9	NCBI	Proteobacteria	Firmicutes	Actinobacteria	Bacteroidetes, Fusobacteria	[42]
	88 (37/51)	54.4 ± 12.8	Korea	V3–V4	Greengenes	Proteobacteria	Firmicutes	Actinobacteria	Bacteroidetes, Cyanobacteria	[43]
	260 (105/155)	56	Korea	V3–V4	SILVA	Firmicutes	Proteobacteria	Actinobacteria	Bacteroidetes, Verrucomicrobia	[44]
	200 (117/83)	63.5 ± 12.5	Korea	V3–V4	Greengenes	Firmicutes	Bacteroidetes	Proteobacteria	Verrucomicrobia, Actinobacteria	[45]
	5 (NA)	NA	UK	V3–V4	SILVA	Proteobacteria	Firmicutes	Actinobacteria	Bacteroidetes, Fusobacteria	[46] ^†^
**RNA**										
Whole blood	14 (12/1)	37.4 ± 10	Japan	V3–V4	Greengenes	Firmicutes	Bacteroidetes	Fusobacteria	Proteobacteria, Actinobacteria	[17]
	49 (38/11)	41.1 ± 10.7	USA	RNA-Seq	PhyloSift	Proteobacteria	Firmicutes	Cyanobacteria	Bacteroidetes, Thermotogae	[47]
Plasma	5 (0/5)	39.4 ± 10.3	UK	RNA-Seq	Kraken/ NCBI	Proteobacteria	Firmicutes	Bacteroidetes	Actinobacteria	[20]

^#^ Age expressed by mean ± SD or median with interquartile range; ^†^ specimens were pretreated by DNase before the DNA extraction; ^‡^ specimens were pretreated by resuscitation process before DNA extraction.

**Table 2:** Blood Bacteriome Dysbiosis Profiles in Infection-Related Diseases.

Subjects (n; Mean Age)	Samples	Dysbiotic Blood Bacteriome of Patients vs. Controls				Other	Interpretation	Ref.
		Diversity			Differential Abundance			
		α-R	α-E	β				
**HIV Infection**								
The Italian study (56): HIV patients before treatment with cART (41; age = 42 (31.5–50.5)) HC (15; age = 29 (24–33))	Plasma DNA	↑	↑	NA	**Family**: ↑*Prevotellaceae*, ↑*Lactobacillaceae*, ↓*Ruminococcaceae*, and ↓*Bacteroidaceae*	NA	Blood dysbiosis in HIV infection might be characterized by an increase in *Prevotellaceae* and *Lactobacillaceae* but a decrease in *Ruminococcaceae* and *Bacteroidaceae*	[38]
The Italian study (41; age = 42 (31.5–50.5)): HIV patients after treatment with cART for 2 years (41) NNRTI (25; age = NA) PI (9; age = NA) INI (7; age = NA) HIV patients before treatment with cART (41)	Plasma DNA	↕	↕	NS	**Family**: ↑*Staphylococcaceae*, ↑*Sphingomonadaceae*, and ↓*Pseudomonadaceae*	NA	cART could modify blood bacteriome with an increase in *Staphylococcaceae* and *Sphingomonadaceae* but a decrease in *Pseudomonadaceae*	[38]
		**Subgroup Analysis**						
		**NNRTI vs. PI and INI**						
		NA	NA	NA	**Family**: ↑*Veillonellaceae*, ↓*Coriobacteriaceae*, and ↓*Peptococcaceae*	**After treated with NNRTI** ↑Endotoxin core antibody **After treated with PI** ↑Intestinal fatty acid-binding protein	HIV infection treated with either NNRTI or PI may lead to an increase in disruption of the gut epithelial barrier, and NNRTI could distinctly modify blood bacteriome by an increase in *Veillonellaceae* but a decrease in *Coriobacteriaceae* and *Peptococcaceae* compared with PI and INI	
The American study (91): HIV patients with cART (40; age = 42 (38–51)) HC (51; age = 42 (35–48))	Plasma DNA	NA	NA	S	**Genus**: ↑*Massilia*, ↑*Haemophilus*, ↑*Veillonella*, ↑*Arthrobacter*, ↑*Fusobacterium*, ↓*Altererythrobacter*, ↓*Cryobacterium*, and ↓*Anaerococcus*	**Validated by in vitro study** ↑TNF-α, ↑IL-1β, and ↑IL-6 from PBMC inoculated with *Massilia* or *Haemophilus* vs. PBMC inoculated with *Anaerococcus*	An increase in *Massilia* and *Haemophilus* in blood bacteriome of HIV infection could lead to chronic systemic inflammation	[53]
The American study (42): HIV patients after receiving influenza vaccines (26; age = NA) High anti-nuclear antibody after vaccination (12; age = 43 (36–54)) Low anti-nuclear antibody after vaccination (14; age = 43 (26–52)) HC after receiving influenza vaccines (16; age = 38 (33–55))	Plasma DNA (before vaccinated)	NA	↕	NS	**Phylum**: ↑Proteobacteria, ↓Actinobacteria, and ↓Firmicutes Genus: ↑*Pseudomonas*	↑anti-nuclear antibody in the HIV patients after receiving influenza vaccines vs. HC	Blood dysbiosis in HIV infection could initiate production of autoantibody, which may be characterized by an increase in Proteobacteria, *Pseudomonas* in particular, and Actinobacteria but a decrease in Firmicutes	[40]
		**Subgroup Analysis**						
		**HIV with high anti-nuclear antibody vs. HIV with low anti-nuclear antibody after vaccinated**						
		NA	↕	NS	**Phylum**: ↑Firmicutes **Genus**: ↑*Staphylococcus* **Species**: ↑*Staphylococcus epidermidis* and ↑*Staphylococcus haemolyticus*	**Validated by in vivo study** ↑anti-dsDNA antibody in mice inoculated with heat-killed *Staphylococcus* vs. mice inoculated with heat-killed *Pseudomonas*	An increased proportion of *Staphylococcus* in blood bacteriome in HIV infection may be involved in the pathophysiology of the autoantibody production after receiving influenza vaccine	
**Sepsis**								
The Polish study (85): Patients with sepsis (62; age = 67) HC (23; age = 59)	Whole blood DNA	↑	NA	S	**Phylum**: ↑Proteobacteria and ↓Actinobacteria **Order**: ↑Rhizobiales, ↑Aeromonadales, ↑Sphingomonadales, ↑Actinomycetales and ↓Bifidobacteriales	NA	Blood dysbiosis in sepsis might be characterized by an increase in Proteobacteria but a decrease in Actinobacteria, Bifidobacteriales in particular	[19]
The Chinese study (51): Post-operative patients with infection (39; age = 54.21 ± 14.01) No sepsis (10; age = 49.4 ± 17.9) Sepsis (18; age = 54.2 ± 12.3) Septic shock, SS (11; age = 58.6 ± 13.4) Controls (12; age = NA) Non-infected (7; age = 49.6 ± 10.5) HC (5; age = NA)	Whole blood DNA	↓	NA	S	NA	**Genus**: *Agrococcus* correlated with Sepsis-related Organ Failure Assessment scores and lactate levels About 80% of blood bacteriome was familiar with gut microbiome (HMP database)	Blood dysbiosis in post-operative patients with infection may originate from the gut microbiome, and *Agrococcus* may play a role in septic progression	[28]
		**Subgroup Analysis**						
		**Septic shock vs. HC**						
		NA	NA	NA	**Phylum**: ↑Bacteroidetes and ↓Actinobacteria **Class**: ↑Flavobacteria, ↑Bacteroidia, ↑Clostridia, ↑Betaproteobacteria, and ↓Gammaproteobacteria **Subclass**: ↓Actinobacteridae	NA	Blood dysbiosis in septic shock may be characterized by an increase in Bacteroidetes but a decrease in Actinobacteria	
The Chinese study (34): Post-operative patients with sepsis (29; age = 55.87 ± 12.71) Sepsis, S (18; age = 54.2 ± 12.3) Septic shock, SS (11; age = 58.6 ± 13.4) HC (5; age = NA)	Neutrophil DNA	↑ (SS) but ↕ (S)	NA	S	**Phylum**: ↑Proteobacteria and ↓Actinobacteria **Class**: ↑Betaproteobacteria, ↑Alphaproteobacteria, and ↓Gammaproteobacteria **Subclass**: ↓Actinobacteridae	About 80% of neutrophil bacteriome were familiar with gut microbiome (HMP database)	Neutrophil bacteriome in post-operative patients with sepsis may originate from the gut microbiome and be characterized by an increase in Proteobacteria but a decrease in Actinobacteria	[28]
The American study (30): PICC-inserted neonates with CLABSI (15; GA = 30 ± 5 weeks) PICC-inserted neonates without CLABSI (15; GA = 32 ± 6 weeks)	Whole blood DNA	↕	↕	S	**CLABSI (n = 3) vs. non-CLABSI (n = 3)** **Family**: ↑*Enterobacteriaceae* **Genus**: ↑*Proteus* and ↑*Staphylococcus*	**Bacteriome of catheter biofilm in CLABSI (n = 15) vs. non-CLABSI (n = 27)** **Genus**: ↑Proteus and ↑Staphylococcaceae	Blood dysbiosis of CLABSI might be associated with ascending infection from catheter biofilm	[54]
**Pregnancy with Pre-Term Delivery**								
The Korean study (41): Pregnant women (21; age = 30.91 ± 4.37) who experienced pre-term delivery at GA 29.67 ± 3.58 weeks Pregnant women (20; age = 31.60 ± 2.91) who experienced term delivery at GA 39.65 ± 1.04 weeks	Plasma-separated blood cell DNA(at labor stage)	↑	NA	S	**Phylum**: ↑Firmicutes, ↑Bacteroidetes, and ↓Proteobacteria **Family**: ↑*Ruminococcaceae*, ↑*Saccharibacteria*, and ↑*Lachnospiraceae* **Genus**: ↑*Bacteroides*, ↑*Lactobacillus*, ↑*Sphingomonas*, ↑*Fastidiosipila*, ↑*Butyricicoccus*, ↑*Methanobrevibacter*, ↓*Delftia*, ↓*Pseudomonas*, ↓*Massilia*, and ↓*Stenotrophomonas*	NA	Blood dysbiosis in pregnant women who had pre-term delivery might be characterized by an increase in Firmicutes and Bacteroidetes but a decrease in Proteobacteria	[55]
The American study (40): Pregnant women (20; age = 22.9 ± 2.7) who later experienced pre-term delivery at GA 29.0 (25.8–30.8) weeks Pregnant women (20; age = 22.7 ± 4.3) who later experienced term delivery at GA 39.6 (39.3–41.1) weeks	Serum DNA (at GA 15–20 weeks)	↑	↑	S	**Phylum**: ↑Proteobacteria, ↑Actinobacteria, ↓Firmicutes, and ↓Bacteroidetes	NA	Blood dysbiosis in mid-trimester pregnant women who had pre-term delivery might be characterized by an increase in Proteobacteria and Actinobacteria but a decrease in Firmicutes and Bacteroidetes	[56]

Age: expressed by mean ± SD or median with interquartile range; Alpha-diversity indices (α): R, richness (either Shannon, phylogenic diversity whole tree, operational taxonomic unit (OTU) counts or Chao1); E, evenness (either Simpson or observed OTU); ↑, significant increase; ↓, significant decrease; ↕, insignificant difference; Beta-diversity indices (β): NS, insignificant difference of Bray-Curtis dissimilarity or unclear separation by principal coordinate analysis (PCoA) plot; S, either significant difference of Bray–Curtis dissimilarity or clear separation by PCoA plot.

**Table 3:** Blood Bacteriome Dysbiosis Profiles in Age-Related Metabolic Diseases.

Subjects (n; Mean Age)	Samples	Dysbiotic Blood Bacteriome Patients vs. Controls				Other	Interpretation	Ref.
		Diversity			Differential Abundance			
		α-R	α-E	β				
**Type 2 Diabetes Mellitus (T2DM) and Obesity**								
The French study (42): HC who, later, were diagnosed with T2DM (14; age = NA) HC (28; age = NA)	Buffy coat DNA	NA	NA	NA	**Phylum**: ↑Proteobacteria and ↓Actinobacteria	↑16s rRNA gene concentration in the HC who, later, were diagnosed with T2DM	Blood dysbiosis characterized by an increase in Proteobacteria and a decrease in Actinobacteria as well as an upsurge in baseline 16s rRNA gene concentration may be involved in the development of T2DM in healthy subjects	[22]
The Chinese study (150): T2DM patients (50; age = 51.64 ± 6.18) HC (100; age = 51.98 ± 8.05)	Plasma DNA	↕	↕	NA	**Order**: ↓Rhodospirillales and ↓Myxococcales **Genus**: ↑*Actinotalea*, ↑*Alishewanella*, ↑*Sediminibacterium*, ↑*Pseudoclavibacter*, ↓*Aquabacterium*, ↓*Xanthomonas*, and ↓*Pseudonocardia*	**Genus**: *Bacteroides* decreased the odds of T2DM (OR 0.331) **Genus**: *Sediminibacterium* increased the odds of T2DM (OR 14.098)	Blood dysbiosis in T2DM might be characterized by a decrease in Rhodospirillales together with Myxococcales, and *Bacteroides* might be a protective factor for T2DM, while *Sediminibacterium* might be a risk factor for T2DM	[18]
The Canadian study (40): Morbid obese patients with T2DM (20; age = 42 ± 9; BMI = 50.9 ± 9.1) Morbid obese patients without T2DM (20; age = 41 ± 9; BMI = 50.2 ± 7.9)	Plasma DNA	↕	↕	NS	**Family**: ↑*Enterobacteriaceae* and ↑*Neisseriaceae* **Genus**: ↑*Escherichia-Shigella* and ↑*Serratia*	↑16s rRNA gene concentration in liver compared with blood in the overall subjects **Genus**: ↑*Bacteroides* in bacteriome of mesenteric adipose tissue in the overall subjects	Blood dysbiosis in morbid obesity with T2DM might be characterized by an increase in *Enterobacteriaceae* and *Neisseriaceae*; in addition, liver might filter microbes in blood derived from gut bacterial translocation	[57]
The German study (75): Morbid obese patients with T2DM (33; age = 52.5 ± 11.3; BMI = 48.8 ± 7.4) Morbid obese patients without T2DM (42; age = 45.2 ± 11.0; BMI = 47.2 ± 5.8)	Plasma DNA	↕	↕	NA	**Genus**: ↑*Tahibacter*, ↓*Delftia*, ↓*Lactobacillus*, and ↓*Lactococcus*	↑Diversity in bacteriome of mesenteric adipose tissues in the overall subjects **Validated in an in vitro study** ↑TNF-α, ↑IL-6, ↑CRP, and ↑LBP from Bacterial-DNA-inoculated adipocytes	Blood dysbiosis in T2DM might be characterized by an increase in *Tahibacter* but a decrease in *Delftia*, *Lactobacillus*, and *Lactococcus*; furthermore, adipose tissues which were exposed to bacteria might initiate chronic systemic inflammation leading to obesity	[58]
The Danish study (29): Obese patients (14; age = 32 (25–58); BMI = 33.4 (30.9–39.8)) HC (15; age = 40 (25–68); BMI = 23.8 (20.7–25.0))	Buffy coat DNA	↕	NA	NA	**Order**: ↑Propionibactereles, ↑Sphingomonadales, and ↑Norcardioides **Family**: ↑*Comamonodaceae* **Genus**: ↓*Enterobacter*	16s rRNA gene concentration in liver correlated with severity of fatty liver (r = 0.44) ↑Diversity in liver bacteriome of the obese patients vs. HC ↑Proteobacteria in liver bacteriome of the obese patients vs. HC	Blood dysbiosis in obesity might be characterized by an increase in Propionibactereles, Sphingomonadales, and Norcardioides; moreover, liver might filter microbes in blood, especially Proteobacteria, and an increase in 16s rRNA gene concentration in liver might play a role in pathogenesis of fatty liver	[30]
**Hypertension**								
The Chinese study (69): Patients with hypertension (41; age = NA) HC (28; age = NA)	Whole blood DNA	↕	↕	S	**Genus**: ↑*Streptococcus*, ↑*Lactobacillus*, ↑*Parabacteroides*, ↑*Helicobacter*, ↓*Stenotrophomonas*, and ↓*Turicibacter*	Blood bacteriome might primarily originate from gastroenteritis, diarrhea, and pneumonia (FAPROTAX database)	Blood dysbiosis in hypertension might be characterized by an increase in *Streptococcus*, *Lactobacillus*, *Parabacteroides*, and *Helicobacter* but a decrease in *Stenotrophomonas* and *Turicibacter*; additionally, blood bacteriome might originate from inflammatory state of gut and lung	[27]
The Chinese study (300): Patients with hypertension (150; age = 47.67 ± 6.02) HC (150; age = 48.13 ± 6.22)	Plasma DNA	↓	↕	NA	**Phylum**: ↑Proteobacteria, ↓Firmicutes, and ↓Bacteroidetes **Genus**: ↑*Sphingomonas*, ↑*Acinetobacter*, and ↓*Staphylococcus*	**Genus**: *Staphylococcus* decreased the odds of hypertension (OR: 0.51) **Genus**: either *Acinetobacter* or *Sphingomonas* increased the odds of hypertension (OR 1.43 and 1.84, respectively)	Blood dysbiosis in hypertension might be characterized by an increase in Proteobacteria but a decrease in Firmicutes and Bacteroidetes; furthermore, *Staphylococcus* might be a protective factor for hypertension while either *Acinetobacter* or *Sphingomonas* might be a risk factor for hypertension	[41]
**Cardiac Diseases**								
The Indian study (41): Patients with cardiac diseases (31; age = 36.55 ± 18.50) VHD (13; age = 31.15 ± 12.19) IHD (11; age = 54.55 ± 6.30) CHD (7; age = 18.29 ± 11.94) HC (10; age = 29.20 ± 11.26)	Whole blood DNA	NA	NA	NA	**Phylum**: ↑Proteobacteria and ↓Firmicutes **Family**: ↓*Staphylococcaceae*	NA	Blood dysbiosis in cardiac diseases might be characterized by an increase in Proteobacteria but a decrease in Firmicutes	[23]
The Indian study (6): Patients with cardiac diseases (3; age = 33 ± 17.35) VHD (1; age = 44) IHD (1; age = 42) CHD (1; age = 13) HC (3; age = 27 ± 3.46)	Whole blood DNA	NA	NA	NA	**Phylum**: ↑Actinobacteria and ↓Proteobacteria **Family**: ↑*Propionibacteriaceae* and ↓*Pseudomonadaceae*	↑16s rRNA gene concentration in all patients vs. HC	Blood dysbiosis in cardiac disease might be characterized by an increase in Actinobacteria but a decrease in Proteobacteria as well as an upsurge in 16s rRNA gene concentration	[15]
The French study (202): Patients with myocardial infarction (99; age = 58.5 (49.9–64.2)) Controls with high cardiovascular risk (103; age = 61.6 (54.9–67.2))	Whole blood DNA	↓	NA	NS	**Family**: ↓*Caulobacteraceae*, ↓*Norcardiaceae* *, and ↓*Aerococcaceae* * **Genus**: ↓*Gordonia* *, ↓*Propionibacterium* *, and ↓*Chryseobacterium* * * Cholesterol-degrading microbes	↑16s rRNA gene concentration in the patients with myocardial infarction vs. controls	Blood dysbiosis in patients with myocardial infarction compared with controls with high cardiovascular risk may be characterized by a decrease in Cholesterol-degrading microbes, including *Norcardiaceae* and *Aerococcaceae,* as well as an increase in 16s rRNA gene concentration	[59]
**Cerebrovascular Accidents**								
The Korean study (398): Patients with acute ischemic stroke (198; age = 63.7 ± 12.5) Good clinical outcomes (159; age = 62.8 ± 11.8) Poor clinical outcomes (39; age = 67.5 ± 14.6) HC (200; age = 63.5 ± 12.5)	EVs DNA (at the onset of stroke)	NA	NA	S	**Phylum**: ↑Proteobacteria and ↓Firmicutes **Order**: ↓Clostridiales, **Family**: ↑*Aerococcaceae* **Genus**: ↑*Flavobacterium*, ↓*Stenotrophomonas*, ↓*Lactobacillus*, ↓*Akkermansia*, and ↓*Mucispirillum*	NA	Blood dysbiosis in acute ischemic stroke might be characterized by an increase in Proteobacteria but a decrease in Firmicutes	[45]
The Korean study (398): Patients with acute ischemic stroke (198; age = 63.7 ± 12.5) Good clinical outcomes (159; age = 62.8 ± 11.8) Poor clinical outcomes (39; age = 67.5 ± 14.6) HC (200; age = 63.5 ± 12.5)	EVs DNA (at the onset of stroke)	**Subgroup Analysis**						[45]
		**Good vs. poor clinical outcomes**						
		NA	NA	NS	**Family**: ↑*Aerococcaceae*, ↑*Microbacteriaceae*, and ↓*Ruminococcaceae* **Genus**: ↑*Anaerococcus*, ↑*Blautia*, ↑*Dialister*, ↑*Propionibacterium*, ↑*Rothia*, and ↓*Prevotella*	NA	An increase in *Aerococcaceae* together with *Microbacteriaceae* and a reduction in *Ruminococcaceae* in blood of patients with acute ischemic stroke might lead to good clinical outcomes	
**Chronic Kidney Disease**								
The American study (40): Patients with chronic kidney disease (20; age = 56 (49–61)) HC (20; age = 44 (39–53))	Buffy coat DNA	↓	NA	NS	**Phylum**: ↑Proteobacteria **Family**: ↑*Enterobacteriaceae* and ↑*Pseudomonadaceae*	Proteobacteria negatively correlated with glomerular filtration rate (r = −0.54)	Blood dysbiosis in chronic kidney disease might be characterized by an increase in Proteobacteria, which may play a role in progression of chronic kidney disease	[31]

*: cholesterol-degrading microbes.

**Table 4:** Blood Bacteriome Dysbiosis Profiles in Oral, Gastrointestinal, and Hepatobiliary Diseases.

Subjects (n; Mean Age)	Samples	Dysbiotic Blood Bacteriome of Patients vs. Controls				Other	Interpretation	Ref.
		Diversity			Differential Abundance			
		α-R	α-E	β				
**Oral Diseases**								
The American study (41): Subjects with tobacco-smoking (20; age = 43 (32–48)) HC (21; age = 38 (30–46))	Plasma DNA	NA	↑	S	**Genus**: ↑*Streptococcus* **Species**: ↑*Streptococcus parasanguinis*, ↑*Streptococcus australis*, and ↑*Streptococcus oligofermentans*	NA	Blood dysbiosis in association with tobacco smoking might be characterized by an increase in *Streptococcus*	[53]
The British study (40): Patients with periodontitis (18; age = 46.61 ± 15.21) HC (22; age = 39.95 ± 13.21)	Whole blood DNA	↕	↕	S	**Phylum**: ↓Candidatus Saccharibacteria **Order**: ↓ Myxococcales	About 70% of blood bacteriomes in both HC and patients with periodontitis were familiar with oral microbiome (Human Oral Microbiome database)	Blood dysbiosis in periodontitis might be characterized by a decrease in Candidatus Saccharibacteria; in addition, blood bacteriome might originate from oral bacteriome	[21]
**Stomach Diseases**								
The Chinese study (84): Patients with gastric cancer (71; age = 59 (52–65)) HC (13; age = NA)	Serum DNA	NA	↓	S	**Genus**: ↑*Haemophilus*, ↑*Acinetobacter*, ↑*Bacteroides*, ↓*Comamonas*, ↓*Sphingomonas*, and ↓*Pseudomonas*	**Genus**: *Enterococcus* correlated with TNM stage and invasion depth (r = 0.42 and 0.43) **Genus**: *Enterococcus* and *Bacteroides* were increased in lymphatic metastasis **Genus**: *Haemophilus* negatively were increased in non-lymphatic metastasis	Blood dysbiosis in gastric cancer might be characterized by an increase in *Haemophilus*, *Acinetobacter*, *Bacteroides*, and *Comamonas* but a decrease in *Sphingomonas* and *Pseudomonas*; furthermore, *Enterococcus* might play a role in progression of gastric cancer	[35]
**Bowel Diseases**								
The Danish study with colon cancer (30; age = 67.6 ± 8.8): Pre-operative patients as controls (30) Post-operative patients (30)	Whole blood DNA	↓	NA	NS	**Post-operative vs. Pre-operative** **Phylum**: ↑Proteobacteria and ↓Actinobacteria **Order**: ↑Pseudomonadales and ↑Enterobacteriales	↓16s rRNA gene concentration in the post-operative patients	Blood dysbiosis in post-operative patients with colon cancer might be characterized by an increase in Proteobacteria but a decrease in Actinobacteria as well as a decline in 16s rRNA gene concentration	[60]
The Chinese study with colon cancer (19; age = 64 (36–81)): Patients before treated with CT as controls (19) Patients who later became drug responders (8; age = 66.5 (53–72)) Patients who later, became drug non-responders (11; age = 62 (36–81)) Patients after being treated with CT (19)	Plasma DNA	↕	↕	NA	**Post-treatment vs. Pre-treatment** **Phylum**: ↑Verrucomicrobia	↓16s rRNA gene concentration in the post-treatment patients	CT could modify blood bacteriome in colon cancer as an increase in Verrucomicrobia while 16s rRNA gene concentration was decreased	[61]
		**Subgroup Analysis**						
		**Patients who later became drug responders vs. drug non-responders**						
		↕	↕	S	**Phylum**: ↑Firmicutes and ↑Fusobacteria	NA	An increase in Firmicutes and Fusobacteria in baseline blood bacteriome of colon cancer could predict the responsiveness of CT	
The Chinese study with colon cancer (20; age = 60 (36–86)): Patients before treatment with CT and DC-CIK as controls (20) Patients who later, became drug responders (13; age = 60 (36–78)) Patients who later, became drug non-responders (7; age = 60 (47–86)) Patients after being treated with CT and DC-CIK (20)	Plasma DNA	↑	↓	NA	**Post-treatment vs. Pre-treatment** **Phylum**: ↑Bacteroidetes	↓16s rRNA gene concentration in the post-treatment patients	CT together with DC-CIK could modify blood bacteriome in colon cancer as an increase in Bacteroidetes while 16s rRNA gene concentration was decreased	[61]
		**Subgroup Analysis**						
		**Patients who later became drug responders vs. drug non-responders**						
		↓	↕	NA	**Genus**: ↑*Lactobacillus*, ↑*Bifidobacterium*, ↑*Enterococcus*, and ↑*Pseudomonas*	**Genus**: *Lactobacillus* correlated with overall survival rate (r = 0.58)	An increase in *Lactobacillus*, *Bifidobacterium*, and *Enterococcus* but a decrease in *Pseudomonas* in baseline blood bacteriome of colon cancer could predict the responsiveness of CT with DC-CIK	
The British study (18): Patients with treated inflammatory bowel diseases (13; age = NA) Crohn’s disease (6; age = NA) Ulcerative colitis (7; age = NA) HC (5; age = NA)	EVs DNA	↕	NA	NS	NA	NA	Blood bacteriome in treated inflammatory bowel diseases might not be different from healthy controls	[46]
**Pancreatobiliary Diseases**								
The Chinese study (62): Patients with acute pancreatitis (50; age = 43.66 ± 11.42) HC (12; age = 29.2 ± 3.8)	Whole blood DNA	↑	NA	S	**Phylum**: ↑Bacteroidetes and ↓Actinobacteria	About 87% of blood bacteriome in both patients and HC was familiar with gut microbiome (HMP database)	Blood dysbiosis in acute pancreatitis might be characterized by an increase in Bacteroidetes but a decrease in Actinobacteria; moreover, blood bacteriome might originate from gut	[24]
The Chinese study (62): Patients with acute pancreatitis (50; age = 43.66 ± 11.42) HC (12; age = 29.2 ± 3.8)	Neutrophil DNA	↑	NA	S	**Phylum**: ↑Bacteroidetes, ↑Firmicutes, ↓Actinobacteria, and ↓Proteobacteria	About 83.1% of neutrophil bacteriome in both patients and HC were familiar with gut microbiome (HMP database)	Neutrophil dysbiosis in acute pancreatitis might be characterized by an increase in Bacteroidetes and Firmicutes but a decrease in Actinobacteria and Proteobacteria; additionally, blood bacteriome might originate from gut	[24]
The Korean study (155): Patients with biliary diseases (67; age = 60.56 ± 13.80) Biliary tract cancer (24; age = 69.8 ± 10.7) Either cholecystitis or cholangitis (43; age = 55.4 ± 15.5) HC (88; age = 54.4 ± 12.8)	EVs DNA	↕	NA	S	**Class**: ↑Clostridia and ↓Gammaproteobacteria	NA	Blood dysbiosis in biliary diseases might be characterized by an increase in Clostridia but a decrease in Gammaproteobacteria	[43]
		**Subgroup Analysis**						
		**Patients with biliary tract cancers vs. HC**						
		↕	NA	S	**Family**: ↑*Bifidobacteriaceae* and ↓*Pseudomonadaceae* **Genus**: ↑*Ralstonia*, ↓*Corynebacterium*, and ↓*Comamonas*	NA	Blood dysbiosis in biliary tract cancers might be characterized by an increase in *Bifidobacteriaceae* but a decrease in *Pseudomonadaceae*	
**Liver Diseases**								
The American study (76): Patients with alcoholic hepatitis (37; age = 44.87 ± 10.76) HC (39; age = 41.9 ± 10.7)	Whole blood DNA	↕	NA	NS	**Phylum**: ↓Bacteroidetes	↑16s rRNA gene concentration in the patients with alcoholic hepatitis vs. HC	Blood dysbiosis in association with alcoholic hepatitis might be characterized by a decrease in Bacteroidetes as well as an increase in 16s rRNA gene concentration	[26]
The morbid obese in Spanish study (37): Patients with cirrhosis (11; age = 48.1 ± 9.3; BMI = 41.9 ± 6.5) Controls without cirrhosis (26; age = 46.2 ± 8.9; BMI = 44.7 ± 6.7)	Buffy coat DNA	↓	NA	NA	**Phylum**: ↑Proteobacteria and ↓Actinobacteria **Class**: ↑Alphaproteobacteria **Family**: ↑*Bradyrhizobiaceae* and ↑*Sphigomonadaceae*	↑16s rRNA gene concentration in the morbidly obese patients with cirrhosis	Blood dysbiosis in morbidly obese patients with cirrhosis compared with morbidly obese patients without cirrhosis might be characterized by an increase in Proteobacteria but a decrease in Actinobacteria as well as an increase in 16s rRNA gene concentration	[25]
The Korean study with NAFLD (76): Obese patients (49; age = 44.6 ± 8.1; BMI = 26.2 ± 1.1) Lean controls (27; age = 46.7 ± 8.3; BMI = 21.8 ± 1.8)	Buffy coat DNA	NA	NA	NS	**Family**: ↑*Succinivibrionaceae* and ↓*Leukonostocaceae*	NA	Blood dysbiosis in obese patients with NAFLD might be characterized by an increase in *Succinivibrionaceae* but a decrease in *Leukonostocaceae* compared with lean patients with NAFLD	[62]
The Korean study (363): Patients with liver disease (162; age = 57.83 ± 10.16) Patients with HCC (79; age = 58.6 ± 9.6) Patients with cirrhosis (83; age = 57.1 ± 10.7) HC (201; age = 57.6 ± 10.4)	Serum DNA	↓	NA	S	**HCC and cirrhosis vs. HC** **Phylum**: ↑Proteobacteria and ↓Firmicutes	NA	Blood dysbiosis in liver diseases (HCC and cirrhosis) might be characterized by an increase in Proteobacteria but a decrease in Firmicutes	[34]
		**Subgroup Analysis**						
		**HCC vs. HC**						
		↓	NA	S	**Genus**: ↑*Staphylococcus*, ↑*Acinetobacter*, ↑*Klebsiella*, ↑*Trabusiella*, ↓*Pseudomonas*, ↓*Streptococcus*, and ↓*Bifidobacterium*	NA	Blood dysbiosis in HCC might be characterized by an increase in *Staphylococcus*, *Acinetobacter*, *Klebsiella*, and *Trabusiella* but a decrease in *Streptococcus* and *Bifidobacterium*	
The Japanese study (80): Patients with cirrhosis (66; age = 70.2 ± 9.9) HCC (48; age = NA) HC (14; age = 37.4 ± 10)	Whole blood RNA	↕	↕	NS	**Order**: ↓ Erysipelotrichales **Family**: ↑*Enterobacteriaceae* and ↓*Rikenellaceae* **Genus**: ↓*Akkermansia*	NA	Blood dysbiosis in cirrhosis might be characterized by a decrease in Erysipelotrichales but an increase in *Enterobacteriaceae* regardless of age	[17]
		**Subgroup Analysis**						
		**HCC vs. HC**						
		NA	NA	NA	**Family**: ↑*Enterobacteriaceae* **Genus**: ↑*Bacteroides* and ↓*Bifidobacterium*	NA	Blood dysbiosis in HCC might be characterized by an increase in *Enterobacteriaceae* regardless of age	
The Chinese study (98): Patients with HBV-DLF (50; age = 48.4 ± 13.2) Patients who died within 28 days after diagnosis (20; age = NA) Patients who survived for 28 days after diagnosed (30; age = NA) Controls (48; age = 50.81 ± 10.53) Patients with HBV-CLF (25; age = 54.4 ± 7.9) HC (23; age = 46.9 ± 13.4)	Plasma DNA	↕	↓	NS	**Phylum**: ↓Actinobacteria and ↓Deinococcus-Thermus **Order**: ↓Enterobacteriales **Family**: ↑*Moraxellaceae* and ↓*Enterobacteriaceae* **Genus**: ↑*Sulfurovum* and ↓*Meiothermus*	↑16s rRNA gene concentration in the patients with HBV (HBV-DLF > HBV-CLF > HC)	Blood dysbiosis in HBV-DLF compared with HBV-CLF and HC might be characterized by a decrease in Actinobacteria and Deinococcus-Thermus; in addition, the liver may filter bacteriome in blood, and its efficacy might depend on liver function	[63]
		**Subgroup Analysis**						
		**HBV-DLF vs. HBV-CLF**						
		↕	↓	NS	**Order**: ↑Campylobacterales and ↓Xanthomonadales **Family**: ↓*Xanthomonadaceae*	NA	Blood dysbiosis in HBV-DLF compared with HBV-CLF might be characterized by an increase in Campylobacterales but a decrease in Xanthomonadales	
		**HBV-DLF vs. HC**						
		↕	↓	NS	**Class**: ↓Alphaproteobacteria **Family**: ↑*Burkholderiaceae* and ↑*Moraxellaceae* **Genus**: ↑*Acinetobacter* and ↑*Comamonas*	NA	Blood dysbiosis in HBV-DLF might be characterized by a decrease in Alphaproteobacteria	
		**Patients who died within 28 days after diagnosis vs. who survived for 28 days after diagnosis**						
		NA	NA	NA	**Family**: ↑*Enterobacteriaceae* and ↓*Prevotellaceae*	NA	Blood dysbiosis in HBV-DLF with poor prognosis might be characterized by an increase in *Enterobacteriaceae* but a decrease in *Prevotellaceae*	

**Table 5:** Blood Bacteriome Dysbiosis Profiles in Neurological Disorders.

Subjects (n; Mean Age)	Samples	Dysbiotic Blood Bacteriome of Patients vs. Controls				Other	Interpretation	Ref.
		Diversity			Differential Abundance			
		α-R	α-E	β				
**Psychiatric Disorders**								
The France study (112): Patients with untreated MDE (56; age = 41.9 ± 11.6) HC (56; age = 41.9 ± 12.7)	Plasma DNA	↕	↕	S	**Phylum**: ↓Fusobacteria and ↓Candidatus Saccharibacteria **Genus**: ↑*Janthinobacterium* and ↓*Neisseria*	NA	Blood dysbiosis in MDE might be characterized by a decrease in Fusobacteria and Candidatus Saccharibacteria	[64]
The France study (56; 41.9 ± 11.6): Patients with MDE after received 3 months of anti-depressive drugs (56) Drug responders (32; age = 40.7 ± 11.99) Drug non-responders (24; age = 43.7 ± 10.99) Patients with MDE before received anti-depressive drugs (56)	Plasma DNA	NA	NA	NA	**Genus**: ↑*Neisseria* and ↓*Janthinobacterium*	NA	Blood dysbiosis in MDE might be reversed by anti-depressive drugs as an increase in *Neisseria* and a decrease in *Janthinobacterium*	[64]
		**Subgroup Analysis**						
		**Responder vs. non-responder before receiving anti-depressants**						
		NA	NA	NA	**Phylum**: ↑Firmicutes, ↓Proteobacteria, and ↓Actinobacteria	NA	MDE patients whose baseline blood has increased Firmicutes and a reduction in Proteobacteria and Actinobacteria may respond to anti-depressive drugs	
The American study (192): Patients with SCZ (48; age = 29.9 ± 5.8) Controls Patients with BP (48; age = 46.5 ± 9.9) Patients with ALS (47; age = 56.4 ± 10.3) HC (49; age = 41.1 ± 10.7)	Whole blood RNA	NA	↑	S	**Phylum**: ↑Planctomycetes and ↑Thermotogae	Composition of blood bacteriome in all groups was similar to gut and oral microbiome (HMP database) Blood bacterial diversity negatively correlated with diversity of T cell population (r = −0.41)	Human blood bacteriome may originate from gut as well as oral bacteriome, and a reduction in diversity of T cell population in SCZ might relate to blood dysbiosis, which was characterized by an increase in Planctomycetes and Thermotogae	[47]
**Neurodegenerative Diseases**								
The Chinese study (90): Patients with Parkinson’s disease (45; age = 68.1 ± 8.0) HC (45; age = 67.9 ± 8.0)	Buffy coat DNA	↕	↕	NS	**Genus**: ↑*Myroides*, ↑*Isoptericola*, ↑*Microbacterium*, ↑*Cloacibacterium*, ↑*Enhydrobacter*, and ↓*Limnobacter*	NA	Blood dysbiosis in Parkinson’s disease might be characterized by an increase in *Myroides*, *Isoptericola*, *Microbacterium*, *Cloacibacterium*, and *Enhydrobacter,* as well as a decrease in *Limnobacter*	[65]
The Chinese study (80): Patients with MSA (40; age = 60.98 ± 6.7) Cerebellar type (17; age = 58.94 ± 7.83) Parkinsonian type (23; age = 62.48 ± 5.48) HC (40; age = 60.88 ± 7.0)	Buffy coat DNA	↕	↕	S	**Genus**: ↑*Bacteroides* and ↓*Leucobacter*	NA	Blood dysbiosis in MSA might be characterized by an increase in *Bacteroides* and a decrease in *Leucobacter*	[66]
		**Subgroup Analysis**						
		**Cerebellar MSA vs. Parkinsonian MSA**						
		NA	NA	NA	**Genus**: ↑*Acinetobacter*, ↓*Blastococcus* and ↓*Bacillus*	NA	Blood dysbiosis in Cerebellar MSA might be characterized by an increase in *Acinetobacter* and a decrease in *Blastococcus* and *Bacillus* compared with Parkinsonian MSA	

**Table 6:** Blood Bacteriome Dysbiosis Profiles in Immunity-Mediated Diseases.

Subjects (n; Mean Age)	Samples	Dysbiotic Blood Bacteriome of Patients vs. Controls				Other	Interpretation	Ref.
		Diversity			Differential Abundance			
		α-R	α-E	β				
**Autoimmune Diseases**								
The American (40): Well-treated SLE patients (21; age = 36.8 ± 9.9) HC (19; age = 34.2 ± 9.4)	Plasma DNA	↕	↕	NS	**Phylum**: ↑Fusobacteria **Genus**: ↓*Paenibacillus*	NA	Blood dysbiosis in well-treated SLE might be characterized by an increase in Fusobacteria	[39]
The American (36): First-degree relatives of SLE patients (18; age = 39.4 ± 12.0) HC (18; age = 38.6 ± 12.4)	Plasma DNA	↓	↓	S	**Phylum**: ↓Firmicutes **Genus**: ↓*Paenibacillus* **Species**: ↑*Thermoanaerobacterium saccharolyticum* and ↑*Lactobacillus iners*	NA	Blood dysbiosis in first-degree relatives of SLE patients might be characterized by a decrease in Firmicutes	[39]
The American (49): SLE patients (19; age = 35 (30–48)) HC (30; age = 43 (32–56))	Plasma DNA	NA	NA	NA	**Genus**: ↑*Planococcus*, ↑*Desulfoconvexum*, ↑*Desulfofrigus*, ↑*Desulfovibrio*, ↑*Draconibacterium*, ↑*Planomicrobium*, ↑*Psychrilyobacter*, ↑*Corynebacterium*, and ↑*Ochrobactrum*	**Validated by in vitro study** ↑TNF-α, ↑IL-1β, and ↑IL-6 from PBMC inoculated with Planococcus	An increase in *Planococcus* in blood bacteriome of SLE could lead to chronic systemic inflammation	[53]
The Chinese (42): Patients with rheumatoid arthritis (28; age = 44.99 ± 9.45) HC (15; age = 50.48 ± 14.05)	PBMCDNA	↕	↕	S	**Phylum**: ↑Candidatus Saccharibacteria and ↓Bacteroidetes	**Genus** *Pelagibacterium* in **family** *Hyphomicrobiaceae* in **order** Rhizobiales correlated with *PARP9* mRNA levels (r = 0.65, 0.66, and 0.60, respectively)	Blood dysbiosis in rheumatoid arthritis may be characterized by an increase in Candidatus Saccharibacteria, but a decrease in Bacteroidetes and an increase in *Pelagibacterium*, *Hyphomicrobiaceae*, and Rhizobiales, might play a role in pathophysiology of rheumatoid arthritis	[32]
The British (30): Patients with untreated rheumatoid arthritis (20; age = NA) Controls (10; age = NA) HC (4; age = NA) Well-treated ankylosing spondylitis (4; age = NA) Well-treated psoriatic arthritis (2; age = NA)	Serum DNA	NA	NA	NA	**Family**: ↑*Lachnospiraceae* **Genus**: ↑*Halomonas*, ↑*Shewanella*, ↓*Corynebacterium 1*, and ↓*Streptococcus*	NA	Blood dysbiosis in rheumatoid arthritis might be characterized by an increase in *Lachnospiraceae*, *Halomonas*, and *Shewanella* but a decrease in *Corynebacterium 1* and *Streptococcus*	[36]
The British (20; age = NA): Patients with rheumatoid arthritis after 3 months of anti-rheumatic treatment (20) Patients with rheumatoid arthritis before treated with anti-rheumatic drugs (20)	Serum DNA	NA	NA	NA	**Family**: ↑*Lachnospiraceae* **Genus**: ↑*Corynebacterium 1*, ↑*Streptococcus*, ↓*Halomonas*, and ↓*Shewanella*	NA	Anti-rheumatic drugs might cause a reversion of blood dysbiosis in rheumatoid arthritis by an increase in *Corynebacterium 1* and *Streptococcus* but a decrease in *Shewanella*; in addition, the persistent increase in *Lachnospiraceae* after treatment might indicate that there might be compensatory effect for blood dysbiosis and could alleviate the disease	[36]
The Taiwanese (28): Patients with psoriasis (20; age = 44.45 ± 16.51) HC (8; age = 49.63 ± 15.16)	EVsDNA	↓	↓	S	**Phylum**: ↓Firmicutes and ↓Fusobacteria **Order**: ↑Bacillales and ↓Lactobacillales **Family**: ↓*Brucellaceae* **Genus**: ↑*Staphylococcus*, ↑*Sphingomonas*, and ↓*Streptococcus* **Species**: ↑*Ralstonia insidiosa*, ↓*Kingella oralis*, and ↓*Aquabacterium parvum*	NA	Blood dysbiosis in psoriasis might be characterized by a decrease in Firmicutes and Fusobacteria	[42]
The French (47): Patients with large vessel arteritis (31; age = 53.77 ± NA) GCA (11; age = 74.08 ± NA) Active (6; age = 77.4 ± NA) Inactive (5; age = 70.1 ± NA) TAK (20; age = 42.6 ± NA) Active (10; age = 43.8 ± NA) Inactive (10; age = 41.4 ± NA) HC (15; age = NA)	Serum DNA	↕	↕	NS	**Class**: ↑Cytophagia and ↑Clostridia **Genus**: ↓*Zooloea* and ↓*Staphylococcus*	NA	Blood dysbiosis in large vessel arteritis might be characterized by an increase in Cytophagia and Clostridia	[37]
		**Subgroup Analysis**						
		**GCA vs. HC**					Blood dysbiosis in both GCA and TAK characterized by an increase in Cytophagia and an upsurge in *Staphylococcus* in TAK might play a role in disease activity	
		NA	NA	NA	**Class**: ↑Cytophagia **Genus**: ↑*Rhodococcus*	NA		
		**TAK vs. HC**						
		NA	NA	NA	**Class**: ↑Cytophagia, ↑Clostridia, and ↑Deltaproteobacteria **Genus**: ↓*Hyphomicrobium* and ↓*Staphylococcus*	NA		
		**GCA vs. TAK**						
		NA	NA	NA	**Family**: ↑*Hyphomonaceae* **Genus**: ↑*Rhodococcus*, and ↓*Cloacibacterium***Species**: ↓*Candidatus aquiluna*	NA		
		**Active TAK vs. inactive TAK**						
		NA	NA	NA	Genus: ↑*Staphylococcus*	NA		
**Rosacea**								
The Korean (40): Patients with rosacea (10; age = NA) HC (30; age = NA)	Whole blood DNA	↕	↕	S	**Family**: ↑*Chromaticeae* and ↑*Fusobacteriaceae* **Genus**: ↑*Rheinheimera*	NA	Blood dysbiosis in rosacea might be characterized by an increase in *Chromaticeae*, *Rheinheimera* in particular, and *Fusobacteriaceae*	[67]
**Asthma**								
The British (10): Patients with asthma (5; age = 39.6 ± 11.7) HC (5; age = 39.4 ± 10.3)	Plasma DNA	↕	↕	NA	**Phylum**: ↑Firmicutes, ↑Bacteroidetes, and ↓Proteobacteria **Order**: ↓Bacteroidales **Class**: ↑Bacilli and ↓Bacteroidia **Family**: ↑*Xanthomonadaceae* **Genus**: ↑*Kocuria* and ↑*Strenotrophomonas*	NA	Blood dysbiosis in asthma might be characterized by an increase in Firmicutes and Bacteroidetes but a decrease in Proteobacteria	[68]
The Korean (450): Patients with asthma (190; age = 48.8 ± 14.6) Steroid naïve (21; age = NA) ICS only (156; age = NA) ICS and OCS (12; age = NA) Unknown (1; age = NA) HC (260; age = 56)	EVsDNA	↑	↓	S	**Phylum**: ↑Bacteroidetes, ↓Actinobacteria, ↓Verrucomicrobia, and ↓Cyanobacteria **Genus**: ↑*Klebsiella*, ↑*Bacteroides*, ↑*Alistipes*, ↑*Subdoligranulum*, ↑*Bifidobacterium*, ↓*Akkermansia*, ↓*Citrobacter*, ↓*Staphylococcus*, and ↓*Micrococcus*	NA	Blood dysbiosis in treated and untreated asthma might be characterized by an increase in Bacteroidetes and Actinobacteria but a decrease in Verrucomicrobia and Cyanobacteria	[44]
		**Subgroup Analysis**						
		**Steroid use vs. steroid naïve**						
		NA	NA	NA	**Genus**: ↓*Staphylococcus* and ↓*Rothia*	NA	Asthma treated with steroids might affect blood bacteriome by a decrease in *Staphylococcus* and *Rothia*	
		**Both ICS and OCS use vs. steroid naïve and ICS only**						
		NA	NA	NA	**Genus**: ↑*Prevotella 9*, ↑*Intestinibacter*, ↑*Lactobacillus*, and ↑*Blautia*	NA	Asthma treated with a combination of ICS and OCS compared with ICS only might affect blood bacteriome by an increase in *Prevotella 9*, *Intestinibacter*, *Lactobacillus*, and *Blautia*	

## Data Availability

Not applicable.

## Abbreviations

16s rRNA	16s ribosomal RNA
ALS	amyotrophic lateral sclerosis
AMI	acute myocardial infarction
cART	combined antiviral therapy
CHD	congenital heart disease
CLABSI	central line bloodstream infection
CLF	compensated liver function
CRP	C-reactive protein
CT	chemotherapy
BMI	body mass index
BP	bipolar disorder
DC-CIK	dendritic cell/cytokine-induced killer cell
DLF	decompensated liver function
EVs	extracellular vesicles
GA	gestational age
GC	giant cell arteritis
HBV	chronic hepatitis B virus infection
HC	healthy controls
HCC	hepatocellular carcinoma
hCV	high cardiovascular risk
HIV	human immunodeficiency virus
HMP	Human Microbiome Project
ICS	inhaled corticosteroids
IHD	ischemic heart disease
IL	interleukin
INI	integrase-inhibitor-based regimen
LBP	LPS-binding protein
LPS	lipopolysaccharide
MDE	major depressive episode
MSA	multiple system atrophy
NAFLD	non-alcoholic fatty liver disease
NCBI	NCBI RefSeq database
NNRTI	non-nucleotide-reverse-transcriptase-inhibitor-based regimen
OCS	oral corticosteroids
PBMC	peripheral blood mononuclear cells
PI	protease-inhibitor-based regimen
PICC	peripherally inserted central catheter
RDP	Ribosomal Database Project
RNA-seq	RNA sequencing
SCZ	Schizophrenia
SLE	systemic lupus erythematosus
TAK	Takayasu’s arteritis
T2DM	type 2 diabetes mellitus
TNF	tumor necrosis factor
V	hypervariable region
VHD	valvular heart disease
